# Development of Functional Performance, Bone Mineral Density, and Back Pain Under Specific Pharmacological Osteoporosis Therapy in an Elderly, Multimorbid Cohort

**DOI:** 10.3390/diagnostics16020297

**Published:** 2026-01-16

**Authors:** Aria Sallakhi, Julian Ramin Andresen, Guido Schröder, Hans-Christof Schober

**Affiliations:** 1 Division of Endocrinology, Rheumatology and Acute Geriatrics, Clinic Ottakring, 1160 Vienna, Austria; 2 Division of Orthopaedics, Department of Orthopaedics and Trauma Surgery, Medical University of Vienna, Währinger Gürtel 18-20, 1090 Vienna, Austria; ramin.andresen@meduniwien.ac.at; 3 Clinic for Orthopaedics and Trauma Surgery, Sana Hospital Bad Doberan, Academic Teaching Hospital of the University of Rostock, Am Waldrand 1, 18209 Hohenfelde, Germany; guido.schroeder1@gmx.net; 4 Practice for Orthopedics and Osteology, OrthoCoast, Hufelandstraße 1, 17438 Wolgast, Germany; hcr.schober@gmx.de

**Keywords:** osteoporosis, functional performance, handgrip strength, Chair Rise Test, tandem stance, bone mineral density, pain, specific pharmacological osteoporosis therapy (SPOT)

## Abstract

**Background/Objectives:** Specific pharmacological osteoporosis therapy (SPOT) is regarded as a key intervention to reduce fracture risk and improve musculoskeletal function. Real-life data, particularly regarding functional muscular outcomes and pain trajectories, remain limited. This study aimed to longitudinally analyze bone mineral density, laboratory parameters, handgrip strength, functional performance, and pain symptoms under guideline-based SPOT. **Methods:** In this monocentric prospective real-life observational study, 178 patients (80.9% women; median age 82 years) with confirmed osteoporosis were followed for a median of four years. All patients received guideline-recommended antiresorptive or osteoanabolic therapy. Analyses included T-scores, 25(OH)D, calcium, handgrip strength, Chair Rise Test (CRT), tandem stance (TS), pain parameters, alkaline phosphatase (AP), HbA1c, fractures, comorbidities, and body mass index (BMI). Time-dependent changes were evaluated using linear mixed-effects models. **Results:** Bone mineral density improved highly significantly (ΔT-score ≈ +0.45 SD; *p* < 0.001), with no differences between therapy groups (antiresorptive vs. osteoanabolic) or BMI categories. Serum 25(OH)D levels increased markedly (Δ ≈ +20 nmol/L; *p* < 0.001), while calcium levels showed a small but highly significant decrease (Δ ≈ −0.047 mmol/L; *p* < 0.001), particularly under antiresorptive treatment. Dominant (Δ ≈ −1.95 kg; *p* < 0.001) and non-dominant handgrip strength (Δ ≈ −0.83 kg; *p* = 0.046) decreased significantly. In contrast, functional performance improved significantly: CRT time decreased by ~1 s (*p* = 0.004), and TS time increased by ~1 s (*p* = 0.007). Back pain decreased highly significantly (Δ ≈ −1.5 NRS; *p* < 0.001), while pain-free walking time (Δ ≈ +38 min; *p* = 0.031) and pain-free standing time (Δ ≈ +31 min; *p* = 0.038) both increased significantly. AP levels decreased significantly (*p* = 0.003), particularly among normal-weight patients. HbA1c changes were not significant. Overall, 73% of patients had at least one major osteoporotic fracture. **Conclusions:** In this real-life cohort, guideline-based specific pharmacological osteoporosis therapy was associated with significant improvements in bone mineral density, vitamin D status, functional performance, and pain-related outcomes. Despite a moderate decline in handgrip strength, balance- and mobility-related functional parameters improved, suggesting preserved or even enhanced functional capacity in daily life. These findings provide real-world evidence on the associations between SPOT, laboratory parameters, functional performance, and pain outcomes in a very elderly and multimorbid population.

## 1. Introduction

Osteoporosis is one of the most prevalent chronic diseases of the aging musculoskeletal system. Approximately 5.6% of the European population is affected [1]. In Germany, the 2023 DVO guideline [2] estimates a prevalence of around six million individuals.

Osteoporosis is characterized by a reduction in bone mass and a deterioration of bone microarchitecture, resulting in impaired skeletal integrity and an increased risk of fractures [3,4]. The condition arises from a multifactorial constellation of risk factors, including advanced age, female sex, endocrine, metabolic and inflammatory disease, medication effects such as glucocorticoids and proton pump inhibitors, prior fragility fractures, falls, immobility, low body weight, and deficiencies in calcium and vitamin D [2]. The typical patient profile includes postmenopausal women with low body mass index, reduced physical activity, and an elevated risk of falls.

Low-energy fractures most commonly occur at the hip, distal radius, and proximal humerus. Additionally, clinically silent vertebral compression fractures are frequently observed [5,6]. These four fracture types are defined as major osteoporotic fractures (MOFs) [2,7,8]. Once a fragility fracture has occurred as a consequence of osteoporosis, the condition is classified as “manifest osteoporosis”. Sacral insufficiency fractures may serve as indicator fractures for a clinically manifest form of the disease [2,9].

Due to demographic aging, an increase in osteoporosis-related fractures and associated healthcare costs is expected in the coming decades [10,11,12,13,14]. Beyond skeletal deterioration, muscular and functional impairments significantly affect the quality of life of individuals with osteoporosis. Reduced muscle strength and limited mobility are associated with an increased risk of falls, thereby aggravating the clinical sequelae of manifest osteoporosis [15]. Common functional impairments include chronic back pain, reduced walking ability, and balance disturbances [15]. These arise primarily from osteoporotic fractures, skeletal deformities, muscular insufficiency, and chronic pain mechanisms. The resulting vicious cycle of pain, avoidance of movement, and consecutive muscle loss further accelerates the osteoporotic process and increases fall risk. The interrelated decline of bone and muscle mass is collectively referred to as osteosarcopenia [16].

In addition to antiresorptive and osteoanabolic pharmacological therapy, physical activity, targeted muscle strengthening, and functional training constitute essential components of modern osteoporosis management [17]. Multiple studies have demonstrated that structured strength and balance training can significantly improve muscle strength, balance, mobility, and bone mineral density, thereby reducing fall risk [2,17,18]. However, real-life data describing how functional parameters such as handgrip strength, balance, pain, and mobility change during specific pharmacological osteoporosis therapy (SPOT) remain limited.

SPOT includes antiresorptive agents such as bisphosphonates and denosumab, which inhibit bone resorption, as well as osteoanabolic agents such as teriparatide and romosozumab, which stimulate bone formation. Both therapeutic approaches have been shown to significantly increase bone mineral density and reduce fracture risk [19,20]. Nevertheless, their effects on pain reduction, functional mobility, and muscular performance remain insufficiently studied in real-life clinical settings.

The aim of this prospective real-life analysis was to evaluate longitudinal changes in bone mineral density, vitamin D and calcium status, handgrip strength, functional performance parameters (Chair Rise Test and tandem stance), pain-free standing and walking time, and back pain intensity over a four-year period under guideline-based specific pharmacological osteoporosis therapy. Based on these findings, the study aims to provide clinically relevant insights into the functional and pain-related effects of SPOT and to derive recommendations for preserving physical performance and mobility.

## 2. Materials and Methods

### 2.1. Ethical Considerations

This analysis was conducted in accordance with the ethical principles of the Declaration of Helsinki. All participants provided informed consent for the pseudonymized use of their data. The study protocol was reviewed and approved by the Ethics Committee of the affiliated university medical faculty (registration number: A 2020–0041).

### 2.2. Study Design and Population

This real-life analysis was conducted as a monocentric prospective observational study at the Osteoporosis Outpatient Clinic of Klinikum Südstadt Rostock. A total of 178 patients with osteoporosis (144 women = 80.9%; 34 men = 19.1%) were included. All collected data were pseudonymized and statistically analyzed. The median age of the study population was 82 years (IQR: 12.8; range: 41–100 years). The median observation period was four years (range: 1–24 years). Inclusion criteria were a confirmed diagnosis of osteoporosis according to the WHO classification (T-score ≤ −2.5 SD) and ongoing guideline-based specific pharmacological osteoporosis therapy (SPOT). Exclusion criteria included lack of follow-up (lost to follow-up), hemi-, para-, or tetraplegia, severe neurological disorders, dementia syndromes, severe peripheral arterial disease (PAD stages IIb, III, and IV), limb amputations, or bone metastases.

### 2.3. Treatment Groups

**Figure 1:**
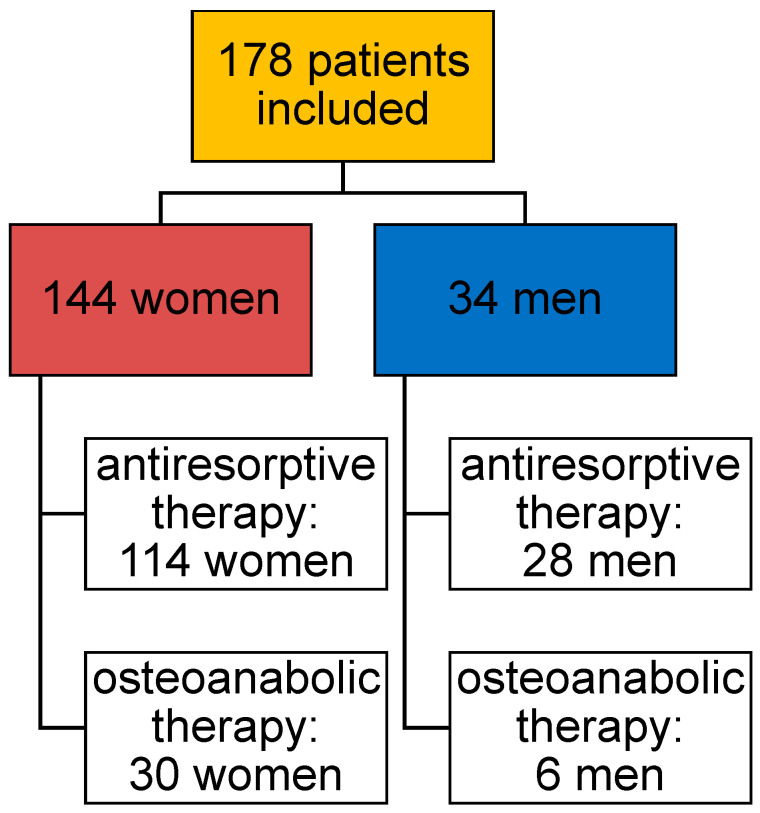
Schematic representation of the study population, including distribution by sex and therapy group (antiresorptive/osteoanabolic).

All patients received guideline-based SPOT in accordance with current osteoporosis treatment recommendations [2]. Two treatment groups were defined: an antiresorptive therapy group and an osteoanabolic therapy group (Figure 1).

Patients in the antiresorptive group received bisphosphonates, the RANK ligand inhibitor denosumab, raloxifene, estradiol or strontium ranelate. This group included both monotherapies and sequential treatment regimens involving transitions between antiresorptive agents. In total, 142 patients were treated with antiresorptive therapy during the course of treatment. Of these, 76 patients received bisphosphonates, 12 denosumab, 1 raloxifene and 1 strontium ranelate (Protelos) as monotherapy, while 52 patients underwent sequential antiresorptive treatment (Table 1, Figure 1).

It should be noted that the production of strontium ranelate (Protelos) was discontinued in August 2017. Patients previously treated with this agent were subsequently switched to an alternative antiresorptive therapy.

Patients in the osteoanabolic group received teriparatide, either as monotherapy or as part of a sequential regimen with preceding or subsequent antiresorptive therapy. Overall, 36 patients received osteoanabolic treatment, including 6 patients treated with teriparatide monotherapy and 30 patients receiving sequential therapy combining an antiresorptive agent and teriparatide. None of the patients received the sclerostin antibody romosozumab (Table 1, Figure 1).

Treatment duration and any medication changes within the therapy groups were documented.

All patients received standardized calcium and vitamin D supplementation throughout the observation period, consisting of 1000 mg calcium daily and 20,000 IU Cholecalciferol once weekly, according to routine clinical practice and guideline recommendations [2]. Supplementation was initiated at baseline and continued during follow-up in all patients. Further calcium and vitamin D prescriptions were carried out by the patient’s general practitioner.

### 2.4. Timepoints

Follow-up was individualized. Baseline was defined as T0 and the last follow-up visit as T3. Intermediate timepoints were defined within each participant’s individual observation period at approximately one-third (T1) and two-thirds (T2) of the total follow-up duration. Time was modelled as a categorial variable (T0–T3/T0&T3) in the linear mixed model analyses.

### 2.5. Measurement Parameters

#### 2.5.1. Bone Mineral Density

The T-score was assessed using dual-energy X-ray absorptiometry (DXA) with a Lunar Prodigy device (GE Healthcare, Boston, MA, USA) at the lumbar spine (L1–L4) and the hip. For each measurement time point, the lowest T-score of the available values was used [2]. Four time points were included for longitudinal evaluation: T0 = start of observation; T1 = 1/3 of the observation period; T2 = 2/3 of the observation period; T3 = end of the observation period. T-scores were documented at all time points. According to current guidelines, T-scores ≤ −2.5 SD were classified as osteoporosis [2].

#### 2.5.2. Vitamin D

Vitamin D status was assessed as part of the osteoporosis-specific laboratory evaluation by measuring the serum concentration of 25-hydroxyvitamin D, 25(OH)D. Four time points were included for longitudinal evaluation: T0 = start of observation; T1 = 1/3 of the observation period; T2 = 2/3 of the observation period; T3 = end of the observation period. In accordance with internationally accepted consensus definitions, 25(OH)D levels < 50 nmol/L were classified as vitamin D deficiency [21,22].

Vitamin D supplementation was systematically implemented as part of the study protocol. All patients received standardized vitamin D supplementation throughout the observation period, consisting of 20,000 IU Cholecalciferol once weekly. Supplementation was initiated at baseline and continued during follow-up in all patients. Further vitamin D prescriptions were carried out by the patient’s general practitioner.

#### 2.5.3. Calcium

Total serum calcium levels were assessed as part of the routine osteoporosis-specific laboratory in this real-life clinical setting. Four time points were included for longitudinal evaluation: T0 = start of observation; T1 = 1/3 of the observation period; T2 = 2/3 of the observation period; T3 = end of the observation period. In accordance with current recommendations, calcium levels <2.12 mmol/L were classified as hypocalcemia [23,24]. Although ionized calcium may provide additional physiological information, total serum calcium is wildly used and accepted for monitoring osteoporosis therapy in routine clinical practice. Measurement of ionized calcium was not part of standard clinical laboratory assessment in this cohort.

Calcium supplementation was systematically implemented as part of the study protocol. All patients received standardized calcium supplementation throughout the observation period, consisting of 1000 mg calcium daily. Supplementation was initiated at baseline and continued during follow-up in all patients. Further calcium prescriptions were carried out by the patient’s general practitioner.

#### 2.5.4. Handgrip Strength

**Figure 2:**
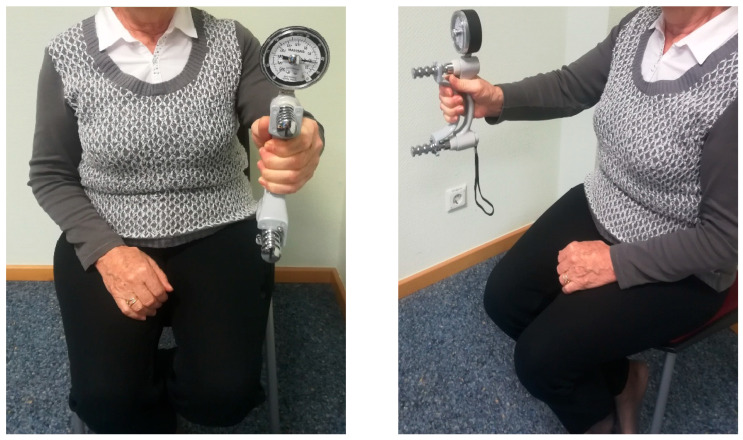
Handgrip strength assessment using a dynamometer in a seated position. Frontal view (**left**) and lateral view (**right**).

Handgrip strength was measured using a Smedley S dynamometer (TMM, Tokyo, Japan, 100 kg) under standardized conditions. Measurements were performed in a seated position with the upper arm adducted, the elbow flexed at 90°, and the forearm and wrist in a neutral position (Figure 2). Four time points were included for longitudinal evaluation: T0 = start of observation; T1 = 1/3 of the observation period; T2 = 2/3 of the observation period; T3 = end of the observation period. At each time point, handgrip strength was measured three times on both the dominant and non-dominant sides. The arithmetic mean of the three measurements was calculated and used as the representative value for each side and time point. According to the current cut-off values of the European Working Group on Sarcopenia in Older People (EWGSOP2) [25], handgrip strength values < 16 kg in women and <27 kg in men were classified as presarcopenia. In the context of osteoporosis, such findings additionally support the suspicion of osteosarcopenia.

#### 2.5.5. Chair Rise Test

**Figure 3:**
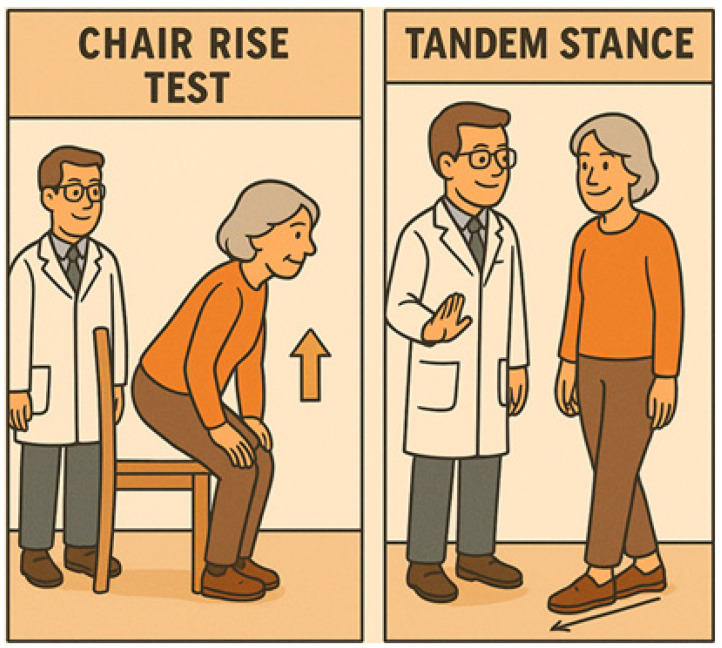
Schematic illustration of the Chair Rise Test (**left**), measured in seconds, and the tandem stance (**right**), measured in seconds, as part of the osteoporosis-specific assessment.

The Chair Rise Test (CRT) was used to assess lower limb strength and sit-to-stand performance (Figure 3, left). It was performed at the beginning (T0) and at the end (T3) of the observation period. The time required to stand up and sit down five times without the use of arms or assistive devices was measured in seconds. According to current guidelines, values > 10 s were interpreted as gait instability, and values > 15 s were considered indicative of (pre-) sarcopenia [2,26].

#### 2.5.6. Tandem Stance

The tandem stance (TS) was used to assess balance impairments and the associated risk of falls (Figure 3, right). Patients positioned their feet in a heel-to-toe alignment along an imaginary line, with the heel of the front foot touching the toes of the back foot. The maximum holding time was measured in seconds (up to 20 s). Two time points were included: T0 = start of the observation period; T3 = end of the observation period. According to current guidelines, values <10 s were interpreted as balance impairment [2,26].

#### 2.5.7. Pain

As part of the routine clinical assessment, patients were systematically asked at each visit, using a standardized patient-reported questionnaire, about the following pain-related parameters: back pain (BP), pain-free walking time (PFWT), and pain-free standing time (PFST) under usual daily life-conditions. PFWT and PFST were self-estimated by the patients as the duration in minutes until pain onset during walking or standing and were not directly observed. Back pain intensity was assessed using the Numerical Rating Scale (NRS; 0–10 points), while PFWT and PFST were recorded in minutes. For this analysis, two pain assessment time points were included: T0 = start of observation; T3 = end of the observation period. These measures reflect patient-reported functional capacity rather than performance-based testing and were therefore considered exploratory.

#### 2.5.8. Alkaline Phosphatase

Alkaline phosphatase (AP) was assessed as a biochemical marker of bone turnover [2,27] as part of the osteoporosis-specific laboratory evaluation. Two time points were included for longitudinal assessment: T0 = start of observation; T3 = end of the observation period.

#### 2.5.9. HbA1c

Glycated hemoglobin (HbA1c) was measured as part of the osteoporosis-specific laboratory evaluation to assess metabolic status. Two time points were included for longitudinal assessment: T0 = start of observation period; T3 = end of the observation period. According to current recommendations, HbA1c values < 5.7% were classified as normal, values between 5.7–6.4% as prediabetes, and values > 6.5% as diabetes mellitus [28].

#### 2.5.10. Fracture Assessment

As part of the clinical data collection, fractures were systematically documented according to their anatomical location. Major osteoporotic fractures (proximal humerus, distal radius, proximal femur, pelvic fractures, and vertebral fractures) [2,7] as well as other fracture types were recorded. To avoid duplicate counting, multiple fractures in the same anatomical region were counted only once per patient.

#### 2.5.11. Comorbidities

Comorbidities were systematically documented for the entire study population. For pathophysiological classification, individual diagnoses were grouped into ten categories: endocrinology/metabolism, cardiovascular, pulmonology, nephrology, gastroenterology/liver/pancreas, immunology/rheumatology, neurology, ophthalmology, psychiatry, and other conditions. For analysis, it was recorded whether each patient had at least one diagnosis within a given category. To avoid duplicate counting, each category was considered only once per patient, regardless of the number of individual diagnoses within that category.

#### 2.5.12. Body Mass Index

As part of the osteoporosis-specific clinical assessment, patients were measured and weighed at each visit, allowing calculation of the Body Mass Index (BMI). Due to small sample sizes in certain BMI categories, the following groupings were applied: underweight = BMI < 18.5 kg/m^2^, normal weight = BMI 18.5–24.9 kg/m^2^, overweight = BMI ≥ 25.0 kg/m^2^. The BMI was used for subgroup analyses in the statistical evaluation.

### 2.6. Outcome Definition and Statistical Hierarchy

The primary outcomes of this real-life observational analysis were longitudinal changes in functional performance parameters, including handgrip strength, Chair Rise Test (CRT) and tandem stance (TS), reflecting clinically relevant aspects of mobility, balance, and muscular function in daily life.

Secondary outcomes comprised changes in bone-related parameters, including bone mineral density (T-score), serum 25-hydroxyvitamin D (25(OH)D), and calcium concentrations.

Pain-related measures, including back pain intensity assessed by the Numerical Rating Scale (NRS) as well as pain-free walking and standing time, were defined as tertiary outcomes.

Additional laboratory parameters (alkaline phosphatase, HbA1c), fracture characteristics, and subgroup analyses were considered exploratory outcomes.

Given the observational real-life design and the multiplicity of investigated endpoints, secondary, tertiary, and exploratory outcomes were analysed in an exploratory manner and should be interpreted with appropriate caution. To address potential type I error inflation, a false discovery rate (FDR)-based sensitivity analysis according to Benjamini and Hochberg was additionally performed for the predefined primary functional outcomes.

### 2.7. Statistical Analysis

Statistical analyses were performed using IBM SPSS Statistics, Version 30 (IBM Corp., Armonk, NY, USA). For the descriptive characterization of the study population, normally distributed variables were presented as mean ± standard deviation (SD) and range, while non-normally distributed variables were reported as median with interquartile range (IQR) and range. Normality was assessed using the Shapiro–Wilk test. Given the non-normal distribution of all parameters, continuous variables were uniformly presented as median with interquartile range (IQR) and range. Categorical variables (sex, treatment group, BMI category) were summarized as absolute and relative frequencies and compared using the chi-square test (χ^2^).

Time-dependent changes in clinical and laboratory parameters (T-score, vitamin D, calcium, handgrip strength, CRT, TS, back pain intensity, PFWT, PFST, and alkaline phosphatase) were analyzed using linear mixed models (LMMs) with a random intercept assigned to each patient. Parameter estimation was performed using restricted maximum likelihood (REML). Fixed effects included time (T0–T3), treatment group (antiresorptive/osteoanabolic), BMI category (underweight, normal weight, overweight), and the interaction terms time × treatment group and time × BMI.

To evaluate statistically significant differences in temporal trends between subgroups, likelihood ratio tests (χ^2^) were applied. Results from the LMM analyses are reported as effect coefficients (β) with corresponding 95% confidence intervals (CI), standard errors (SE), and *p*-values. Effect sizes were calculated according to Cohen’s d to assess clinical relevance (small effect ≥ 0.2, medium effect ≥ 0.5, large effect > 0.8). A *p*-value < 0.05 was considered statistically significant, and values <0.001 were interpreted as highly significant.

Sensitivity analyses were performed to assess the robustness of the main findings. First, analyses were repeated after restricting the cohort to patients receiving monotherapy only (antiresorptive monotherapy or osteoanabolic monotherapy), excluding sequential treatment regimes. Due to reduced sample size, these analyses were considered exploratory and focused on consistency of effect direction rather than statistical significance.

### 2.8. Graphical Presentation

Results were displayed in both tabular and graphical formats. Graphical illustrations were generated using Microsoft Excel (Version 2509). In tables, graphical figures and text, data were reported according to their distribution (mean ± SD + range or median + IQR + range).

## 3. Results

### 3.1. Changes in Bone Mineral Density Under SPOT

Bone mineral density (T-score) improved highly significantly under SPOT at both the lumbar spine (L1–L4) and the proximal femur. The mean T-score increased by approximately 0.45 SD between T0 and T3 (β = 0.445; 95% CI = 0.263 to 0.627; SE = 0.093; *p* < 0.001; d ≈ 0.72). At baseline (T0), the median T-score was −3.5 SD (IQR: 0.96; range: −6.0 to −2.5 SD), increasing to −3.10 SD at T3 (IQR: 1.20; range: −4.95 to −0.90 SD).

Subgroup analyses revealed no significant differences in T-score development between the antiresorptive and osteoanabolic treatment groups (χ^2^(3) = 2.36; *p* = 0.501). Likewise, no significant differences were observed across BMI categories, including underweight, normal-weight, and overweight patients (χ^2^(6) = 7.40; *p* = 0.285).

**Figure 4:**
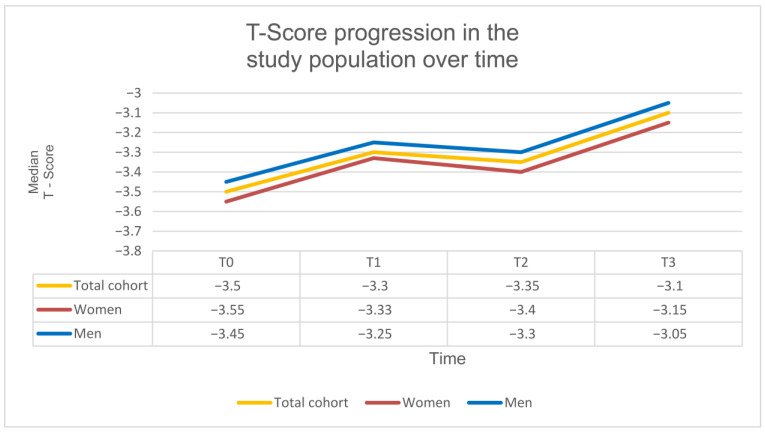
T-score over the course of SPOT. Progression of T-score at time points T0–T3 in the total cohort and stratified by sex.

Overall, these findings demonstrate a clear and highly significant increase in T-score values over the course of guideline-based SPOT (Table 2, Figure 4).

### 3.2. Changes in Vitamin D Levels Under SPOT

Under standardized vitamin D supplementation vitamin D levels (25(OH)D) improved highly significantly over time. The mean 25(OH)D concentration increased by approximately 20 nmol/L between T0 and T3 (β = 19.87; 95% CI = 12.05 to 27.69; SE = 3.99; *p* < 0.001; d ≈ 0.83). At baseline (T0), the mean 25(OH)D level was 69.4 nmol/L (IQR: 38.2; range: 11.0 to 156.0 nmol/L), increasing to 83.5 nmol/L at (IQR: 32.3; Range: 31.9–148.0 nmol/L).

Subgroup analyses revealed no significant differences in 25(OH)D development between the antiresorptive and osteoanabolic treatment groups (χ^2^(3) = 3.28; *p* = 0.351). Likewise, no significant differences were observed between underweight, normal-weight, and overweight patients (χ^2^(6) = 2.23; *p* = 0.898).

**Figure 5:**
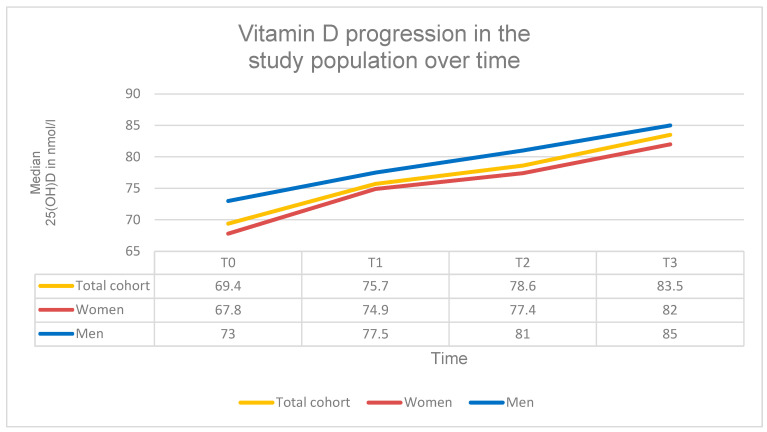
Serum 25(OH)D levels. Progression of 25(OH)D concentrations (nmol/L) at time points T0–T3 in the total cohort and stratified by sex.

At T0, 38 patients (21.3%) presented with vitamin D deficiency. Over time, the number of deficient patients decreased markedly: 13 cases at T1 (7.3%), 12 cases at T2 (6.7%), and only 4 cases at T3 (2.2%). These findings demonstrate a clear and highly significant improvement in vitamin D status over the course of guideline-based vitamin D supplementation during SPOT (Table 3, Figure 5).

### 3.3. Changes in Calcium Levels Under SPOT

Under standardized calcium supplementation calcium levels decreased highly significantly. The mean serum calcium concentration declined by approximately 0.0470 mmol/L between T0 and T3 (β = −0.0470; 95% CI = −0.0737 to −0.0203; SE = 0.0136; *p* = 0.0005; d = 0.32). At baseline (T0), the median calcium level was 2.40 mmol/L (IQR: 0.16; range: 1.92–3.30 mmol/L), decreasing slightly to 2.39 mmol/L at T3 (IQR: 0.14; range: 2.14–3.10 mmol/L).

Subgroup analyses showed a significant difference in calcium development between the antiresorptive and osteoanabolic treatment groups (χ^2^(3) = 15.43; *p* = 0.0015). In the antiresorptive therapy group, calcium levels decreased highly significantly by approximately 0.06 mmol/L between T0 and T3 (β = −0.064; 95% CI = −0.0934 to −0.0346; SE = 0.015; *p* < 0.001), reaching 2.35 mmol/L at T3 (IQR: 0.17; range: 1.80–2.85). In contrast, the osteoanabolic therapy group showed a non-significant increase of approximately 0.021 mmol/L (β = +0.021; 95% CI = −0.0221 to 0.0641; SE = 0.022; *p* = 0.352), reaching 2.38 mmol/L at T3 (IQR: 0.09; range: 2.27–2.53).

No significant differences in calcium development were found between underweight, normal-weight, and overweight patients (χ^2^(6) = 8.66; *p* = 0.194). The incidence of patients with hypocalcemia was low: one case at T0 (0.6%), two cases at T2 (1.1%), and three cases at T3 (1.7%).

**Figure 6:**
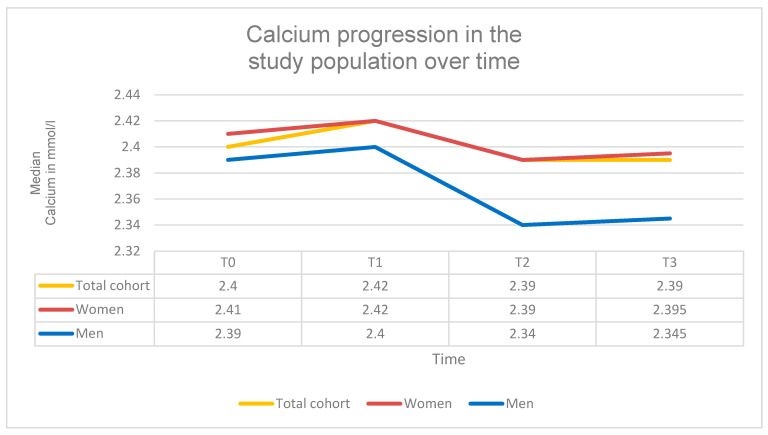
Serum calcium levels. Progression of serum calcium concentrations (mmol/L) at time points T0–T3 in the total cohort and stratified by sex.

Despite continuous calcium supplementation, calcium levels showed a significantly decreasing trend over time in the total study population (Table 4, Figure 6).

### 3.4. Changes in Handgrip Strength Under SPOT

Under SPOT, mean dominant handgrip strength decreased highly significantly by approximately 2 kg (β = −1.954; 95% CI = −2.793 to −1.115; SE = 0.428; *p* < 0.001; d ≈ 0.31). At baseline (T0), dominant handgrip strength was 26.0 kg (IQR: 10; range: 9.0–62.0 kg), decreasing to 24.0 kg at T3 (IQR: 13.0; range: 6.0–56.0 kg). Subgroup analyses revealed no significant differences in dominant handgrip strength development between treatment groups (χ^2^(3) = 0.60; *p* = 0.879) or BMI categories (χ^2^(6) = 5.39; *p* = 0.496). These findings demonstrate a highly significant decline in dominant handgrip strength during SPOT.

**Figure 7:**
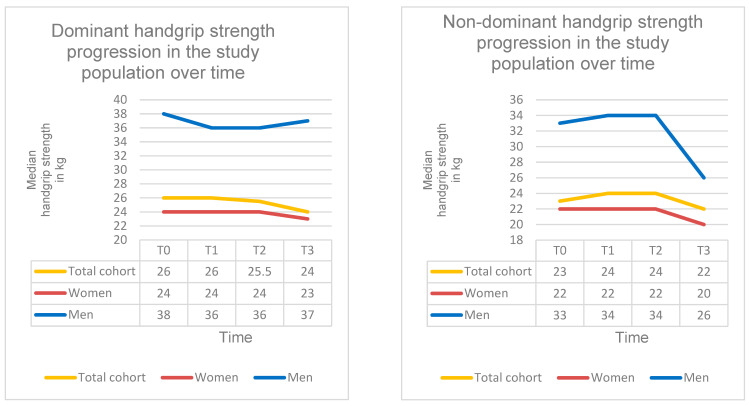
Handgrip strength over the course of SPOT. Progression of handgrip strength at time points T0–T3 in the total cohort and stratified by sex.

Mean non-dominant handgrip strength also decreased significantly by approximately 0.8 kg over the course of SPOT (β = −0.830; 95% CI = −1.643 to −0.0166; SE = 0.415; *p* = 0.046; d = 0.18). At T0, non-dominant handgrip strength was 23.0 kg (IQR: 8.0; range: 4.0–57.0 kg), while the value at T3 was 22.0 kg (IQR: 10.0; Range: 8.0–48.0 kg). Subgroup analyses showed no significant differences in non-dominant handgrip strength development between treatment groups (χ^2^(3) = 1.52; *p* = 0.678) or BMI categories (χ^2^(6) = 2.21; *p* = 0.900). These findings demonstrate a significant decline in non-dominant handgrip strength during SPOT (Table 5, Figure 7).

### 3.5. Changes in Chair Rise Test Under SPOT

Mean CRT time decreased significantly by approximately 1 s during SPOT (β = −0.98; 95% CI = −1.627 to −0.333; SE = 0.33; *p* = 0.004; d ≈ 0.30). At baseline (T0), CRT time was 11.0 s (IQR: 3.0; range: 7–20 s), improving to 10.0 s at T3 (IQR: 3.0; range: 6–18 s). The incidence of pathological findings (>15 s) decreased from 7% at T0 to 4% at T3.

Subgroup analyses revealed no significant differences in CRT development between treatment groups (χ^2^(1) = 0.04; *p* = 0.842) or BMI categories (χ^2^(2) = 0.72; *p* = 0.698).

**Figure 8:**
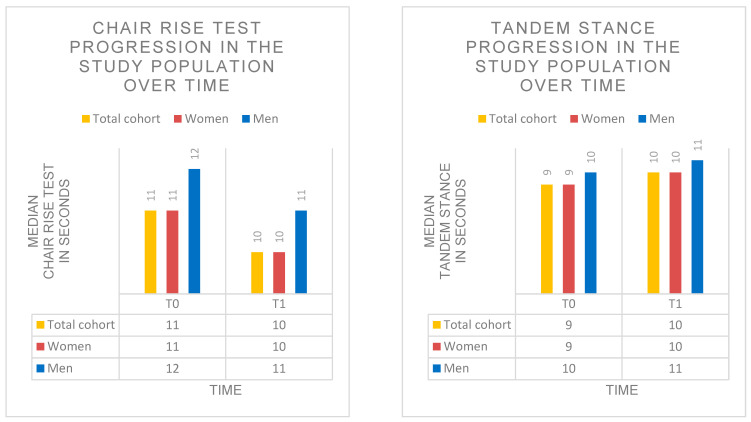
Chair Rise Test and tandem stance over the course of SPOT. Progression of CRT and TS performance at time points T0–T3 in the total cohort and stratified by sex.

Overall, these findings indicate a significant improvement in balance as well as lower-limb strength and sit-to-stand performance during guideline-based SPOT (Table 6, Figure 8).

### 3.6. Changes in Tandem Stance Under SPOT

Mean TS time increased significantly by approximately 1 s during SPOT (β = +0.84; 95% CI = 0.232 to 1.448; SE = 0.31; *p* = 0.007; d ≈ 0.29). At baseline (T0), TS time was 9.0 s (IQR: 4.0; range: 2–20 s), increasing to 10.0 s at T3 (IQR: 5.0; range: 3–20 s). The incidence of pathological findings (<10 s) decreased from 24% at T0 to 15% at T3.

Subgroup analyses showed no significant differences in TS development between treatment groups (χ^2^(1) = 0.02; *p* = 0.889) or BMI categories (χ^2^(2) = 0.05; *p* = 0.974).

Overall, these findings indicate a significant improvement in balance and a reduction in fall risk during guideline-based SPOT (Table 6, Figure 8).

Given the number of investigated endpoints, outcomes were hierarchically classified into primary, secondary, tertiary and exploratory endpoints. Primary outcomes included functional performance parameters (Chair Rise Test, tandem stance and handgrip strength. Secondary and tertiary outcomes were considered exploratory. To address the risk of type I error inflation, a false discovery rate (FDR)-based sensitivity analysis according to Benjamini and Hochberg was additionally performed for the primary functional outcomes. All primary functional outcomes remained statistically significant after FDR correction, FDR q < 0.05, supporting the robustness of the main findings.

In sensitivity analyses restricted to monotherapy regimes, changes in functional performance (Chair Rise Test, tandem stance and handgrip strength) showed similar direction compared with the main analysis, although statistical significance was not consistently reached due to limited sample size. No reversal of effects was observed.

### 3.7. Changes in Pain Parameters Under SPOT

Mean back pain intensity decreased highly significantly during SPOT, with a reduction of approximately −1.5 NRS points (β = −1.46; 95% CI = −2.322 to −0.598; SE = 0.44; *p* < 0.001; d ≈ 0.61). At baseline (T0), back pain intensity was 5.0 NRS (IQR: 3.0; range: 0–10), decreasing to 3.0 NRS at T3 (IQR: 3.0; range: 0–9). Subgroup analyses showed no significant differences in the development of back pain intensity between the antiresorptive and osteoanabolic treatment groups (χ^2^(1) = 0.17; *p* = 0.678).

Mean pain-free walking time increased significantly by approximately 38 min over the course of SPOT (β = +37.55; 95% CI = 3.52 to 71.58; SE = 17.36; *p* = 0.031; d ≈ 0.42). At T0, pain-free walking time was 60.0 min (IQR: 90.0; range: 0–500), rising to 99.0 min at T3 (IQR: 68.0; range: 15–200). No significant differences were observed between treatment groups regarding the development of pain-free walking time (χ^2^(1) = 0.27; *p* = 0.606).

Mean pain-free standing time increased significantly by approximately 31 min during SPOT (β = +31.2; 95% CI = 2.78 to 59.62; SE = 14.5; *p* = 0.038; d ≈ 0.45). At T0, pain-free standing time was 45.0 min (IQR: 60.0; range: 0–400), increasing to 78.0 min at T3 (IQR: 55.0; range: 22–160). Subgroup analyses revealed no significant differences between treatment groups in the progression of pain-free standing time development (χ^2^(1) = 0.31; *p* = 0.578).

Sensitivity analyses were performed excluding extreme values above the 95th percentile of pain-free walking and standing time distributions. After exclusion, improvements remained directionally consistent, although effect sizes were attenuated, indicating that the observed changes were not driven solely by extreme values.

In an exploratory sex-stratified analysis, changes in pain-related outcomes were comparable between women and men. No relevant sex-specific differences in pain trajectories were observed.

It should be noted that a high proportion of the cohort had experienced major osteoporotic fractures, predominantly vertebral fractures, which are known to substantially influence pain perception and functional capacity (Table 9).

**Figure 9:**
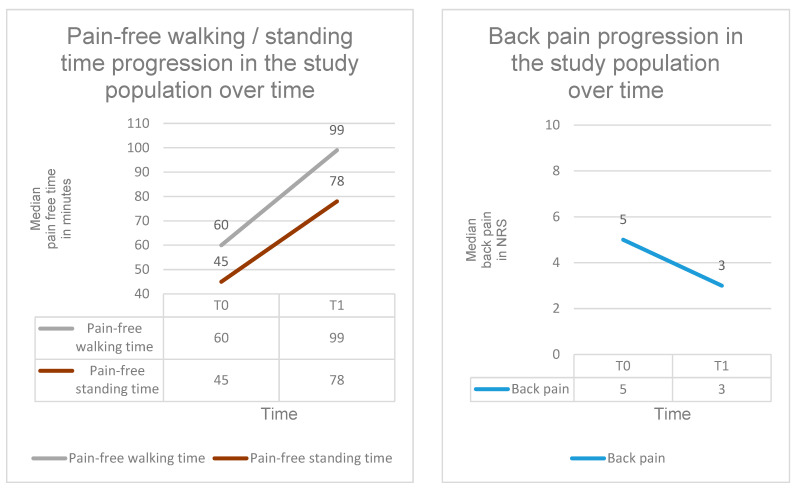
Pain-free walking time, pain-free standing time and back pain intensity over the course of SPOT. Progression of PFWT, PFST, and back pain intensity at time points T0–T3 in the total cohort.

Overall, these results demonstrate a (highly) significant and clinically meaningful reduction in pain among patients undergoing SPOT (Table 7, Figure 9). Furthermore, the findings indicate improvements in functional capacity, activity tolerance, and mobility over the course of treatment.

### 3.8. Changes in Alkaline Phosphatase Under SPOT

Alkaline phosphatase (AP) levels decreased significantly during SPOT. The mean AP concentration declined by approximately 0.14 µkat/L between T0 and T3 (β = −0.137; 95% CI = −0.227 to −0.0468; SE = 0.046; *p* = 0.003; d = 0.31). At baseline (T0), the AP level was 1.34 µkat/L (IQR: 0.57; range: 0.29–4.42 µkat/L), decreasing to 1.28 µkat/L at T3 (IQR: 0.51; range: 0.36–3.48 µkat/L).

Subgroup analyses revealed no significant differences in AP development between the antiresorptive and osteoanabolic treatment groups (χ^2^(1) = 0.97; *p* = 0.33). A significant difference was observed between BMI categories (χ^2^(2) = 6.73; *p* = 0.035). Normal-weight patients showed a significant reduction in AP levels of approximately 0.25 µkat/L (β = −0.254; 95% CI = −0.379 to −0.129; SE = 0.064; *p* < 0.001). In contrast, the decreases in the underweight and overweight groups were not statistically significant.

**Figure 10:**
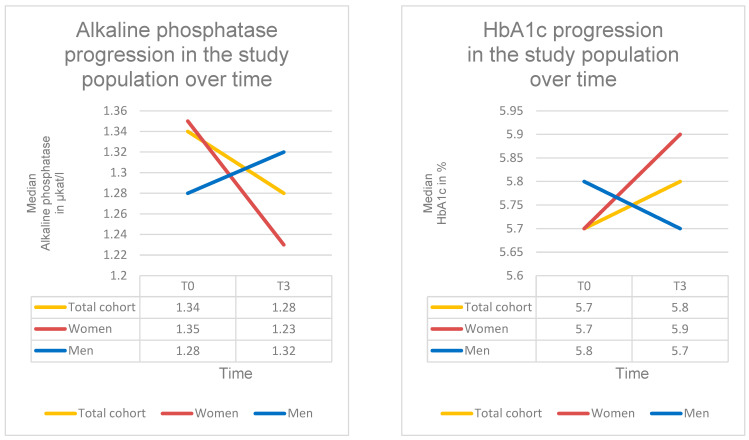
Serum alkaline phosphatase and HbA1c levels over the course of SPOT. Progression of AP concentrations (µkat/L) and HbA1c values (%) at time points T0–T3 in the total cohort and stratified by sex.

Overall, these findings indicate a clear and statistically significant reduction in AP levels during guideline-based SPOT (Table 8, Figure 10).

### 3.9. Changes in HbA1c Under SPOT

At baseline (T0), the median HbA1c level was 5.70% (IQR: 0.77; range: 4.80–10.0%), and 5.80% at T3 (IQR: 0.36; range: 4.90–9.00%). While the descriptive median showed a slight increase from T0 to T3, the linear mixed model analysis demonstrated a small, non-significant decrease in HbA1c of approximately 0.06% during SPOT (β = −0.063; 95% CI = −0.318 to 0.192; SE = 0.130; *p* = 0.631; d = 0.14).

Overall, 85 patients (48%) had HbA1c levels < 5.7%, 68 patients (38%) had values between 5.7% and 6.4% consistent with prediabetes, and 25 patients (14%) had HbA1c levels > 6.5%, indicating diabetes mellitus (Table 8, Figure 10).

### 3.10. Changes in Height, Weight and Body Mass Index Under SPOT

**Figure 11:**
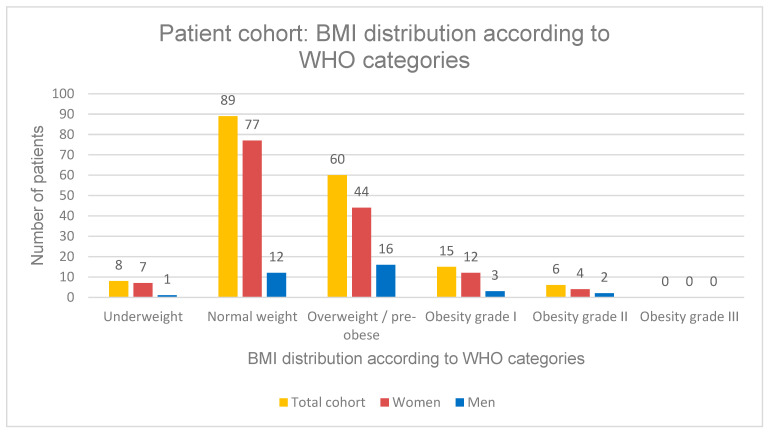
Schematic overview of the patient cohort by BMI according to WHO categories.

The study population had a median height of 1.63 m (IQR: 0.10; range: 1.45–1.93 m), a median body weight of 66.0 kg (IQR: 17.2; range: 35.9–120.0 kg), and a median body mass index (BMI) of 24.8 kg/m^2^ (range: 16.1–39.9 kg/m^2^). According to BMI classification, 8 patients (4.5%) were underweight (BMI < 18.5 kg/m^2^), 89 patients (50.0%) had normal weight (BMI 18.5–24.9 kg/m^2^), 60 patients (33.7%) were overweight or pre-obese (BMI 25.0–29.9 kg/m^2^), 15 patients (8.4%) had obesity grade I (BMI 30.0–34.9 kg/m^2^), and 6 patients (3.4%) had obesity grade II (BMI 35.0–39.9 kg/m^2^). No patients with obesity grade III (BMI > 40 kg/m^2^) were present in the study cohort (Figure 11).

### 3.11. Fractures in the Study Population

Of the 178 patients in the total cohort, only 24 patients (13.5%) had no clinically documented fractures. Furthermore, 130 patients (73.0%) had sustained at least one major osteoporotic fracture (MOF). These included fractures of the proximal humerus, distal radius, proximal femur, pelvis and vertebral fractures of the cervical, thoracic, and lumbar spine, as well as sacral fractures. In addition, 91 patients (51.1%) experienced at least one other type of fracture, such as rib, sternal, hand, or foot fractures (Table 9).

### 3.12. Comorbidities

Of the 178 osteoporosis patients in the total cohort, 137 (77.0%) had at least one relevant comorbidity. Comorbidities were classified into ten major categories. Each category was counted only once per patient, regardless of the number of individual diagnoses within it. The most frequent category was endocrinology/metabolism, represented by 106 patients (59.6%). A detailed overview of patient comorbidities is provided in Table 10.

## 4. Discussion

### 4.1. Diagnostics

In the presence of clinical suspicion of osteoporosis, all patients received a DXA assessment in accordance with current guidelines [2,29]. T-score values under ≤−2.5 SD confirmed the diagnosis of osteoporosis [2,3]. The high proportion of major osteoporotic fractures (73.0%), predominantly vertebral compression fractures, reflects an advanced stage of manifest osteoporosis in this real-life cohort and underscores the high disease burden of the study population.

### 4.2. Bone Mineral Density

Under SPOT, median T-scores increased by 0.45 SD over the four-year observation period. This longitudinal improvement is consistent with the well-established effects of antiresorptive therapies, which act through inhibition of osteoclast activity [30], and osteoanabolic agents, which stimulate osteoblast function [31]. Both therapeutic classes are known to increase bone mineral density and reduce fracture risk [19,20,32]. The observed attenuation of T-score improvement at later time points may reflect several factors typical of very elderly real-life cohorts, including limited anabolic bone reserve at advanced age, therapy sequencing or pauses, regression-to-the-mean effects, and survivorship bias.

No significant differences in T-score trajectories were observed between antiresorptive and osteoanabolic treatment classes, consistent with the findings of Vilaca et al. [33], indicating that the medication selection should be based on individual risk profiles and current guideline recommendations [2].

The observed association between SPOT and improved bone mineral density in this cohort confirms the effectiveness of SPOT even in a very elderly and multimorbid patient population [32].

### 4.3. Vitamin D and Calcium

Vitamin D plays a central role in calcium homeostasis, bone mineralization, and neuromuscular function [34]. Deficiency promotes secondary hyperparathyroidism, accelerated bone resorption, and an increased fracture risk [35,36,37]. At baseline, 21.3% of patients presented with vitamin D deficiency, a prevalence slightly lower than reported for the general population [38]. During follow-up under SPOT, serum 25(OH)D levels increased by approximately 20 nmol/L, and the proportion of patients with vitamin D deficiency declined markedly to 2.2%. The observed increase in serum 25(OH)D levels must be interpreted in the context of standardized vitamin D supplementation, which was routinely administered to all patients throughout the observation period. Accordingly, the improvement in vitamin D status primarily reflects effective supplementation under routine care rather than a direct effect of SPOT. Nevertheless, adequate vitamin D availability is a prerequisite for optimal response to both antiresorptive and osteoanabolic therapies [39,40,41] and may indirectly contribute to improvements in bone mineral density and functional outcomes. Vitamin D and calcium are essential components of osteoporosis management according to current guidelines [2]. Moreover, combined supplementation has been shown to contribute to improvements in bone mineral density [36].

Antiresorptive agents are known to lower serum calcium levels [42], whereas teriparatide tends to increase them [43]. This class-specific pattern was also observed in the present cohort, reflected by small but characteristic changes in calcium concentrations. The slight decline in serum calcium levels despite continuous calcium supplementation, particularly in patients receiving antiresorptive therapy, is consistent with reduced bone resorption and increased skeletal calcium retention. Importantly, clinically relevant hypocalcaemia remained rare, underscoring the safety of routine supplementation in this elderly cohort. Because hypocalcemia may limit therapeutic response and increase the risk of adverse effects [44], adequate calcium availability is essential.

The use of total serum calcium instead of ionized calcium reflects routine clinical practice in a real-life setting. While ionized calcium may be more sensitive in specific metabolic conditions, total calcium measurement is sufficient for therapy monitoring and safety assessment in most osteoporosis patients.

Overall, these findings underscore the importance of continuous monitoring and sufficient supplementation of vitamin D and calcium as integral components of guideline-based SPOT [2].

### 4.4. Handgrip Strength and Functional Performance Parameters

Reduced handgrip strength is a key diagnostic criterion for sarcopenia and is associated with impaired physical function, an increased risk of falls and fractures, morbidity and mortality [25,45]. In patients with osteoporosis, the combined presence of reduced bone and muscle mass/function is referred to as osteosarcopenia [16]. Despite significant associations between SPOT and improvements in bone mineral density as well as performance in functional tests (CRT, TS), handgrip strength declined over the time in this cohort, consistent with previous observations by Schröder et al. [32]. Given that reduced handgrip strength is strongly associated with systemic diseases and overall health status [46], the observed decline in this cohort likely reflects age-related physiological changes and multimorbidity rather than insufficient treatment efficacy. Differences in handgrip strength trajectories, particularly of the dominant hand, may be influenced by factors beyond osteoporosis therapy itself. Dominant handgrip strength is particularly influenced by habitual daily use and age-related functional decline. In very elderly patients, reduced use due to pain, fractures, osteoarthritis, or neurological comorbidities may disproportionately affect dominant hand strength, largely independent of pharmacological effects.

In contrast, CRT and TS performance improved by approximately one second each, indicating enhanced functional capacity, better lower-limb strength and balance. While the observed changes in CRT and TS were small in absolute terms (approximately one second), their clinical relevance should be interpreted in the context of established functional thresholds. In geriatric populations, crossing cut-off values such as a CRT > 15 s or a TS < 10 s are associated with increased fall risk and functional dependency. In this very elderly and multimorbid cohort, even modest shifts toward safer functional ranges may therefore represent clinically meaningful stabilization or improvement rather than trivial change. Given the advanced age and the high burden of comorbidity, the prevention of further functional decline may be considered a relevant therapeutic goal alongside absolute functional gains.

Although several studies have described potential positive muscular effects of anti-osteoporotic agents [47,48,49,50,51], the evidence remains heterogeneous [52].

Importantly, sensitivity analyses excluding sequential therapies yielded comparable directions of change in functional outcomes. It is assumed that the observed associations are not driven solely by heterogenous treatment sequences. However, these analyses were underpowered and therefore interpreted as supportive rather than confirmatory.

Taken together, these findings suggest that handgrip strength alone may be an unsuitable parameter for diagnosing or monitoring osteoporosis in adults over 60 years of age [53]. In this osteoporotic population, functional performance measures may provide a more accurate reflection of therapy-related changes and overall musculoskeletal health.

### 4.5. Pain

Under SPOT, a clinically meaningful reduction in back pain was observed (−1.5 NRS points), accompanied by substantial increases in pain-free walking time (+38 min) and pain-free standing time (+31 min). These findings are consistent with the results reported by Voßmann [54] and correspond well with the functional improvements demonstrated in CRT and TS performance.

Pain-free walking and standing time were assessed using patient-reported estimates, which may be subject to recall and anchoring bias, particularly in an elderly cohort. Accordingly, these parameters were analysed as exploratory outcomes. They should be interpreted as patient-reported functional estimates rather than objective performance measures. Sensitivity analyses excluding extreme values over the 95th percentile yielded consistent directions of change, supporting the robustness of the observed associations.

Pain reduction occurred independently of the therapeutic class and is supported by existing literature describing the analgesic effects of specific pharmacological osteoporosis therapy [55].

The high prevalence of MOF, particularly vertebral fractures, represents a key characteristic of this real-life cohort and a major determinant of pain and functional impairment. Vertebral fractures are known to contribute to chronic back pain, reduced mobility and balance deficits. Accordingly, the observed trajectories of pain and functional parameters should be interpreted in the context of a fracture-enriched population. Given the observational design, the present analysis does not allow disentanglement of therapy-associated effects from fracture-related recovery, adaptation, or survivorship effects. Rather than attributing changes in pain or function causally to pharmacological therapy, the findings describe longitudinal associations under routine clinical care in a cohort with advanced disease severity.

As pain management in osteoporosis requires a multimodal approach, SPOT represents an important, but not exclusive, component within a comprehensive, multidisciplinary treatment strategy [2,55,56,57].

Future studies should stratify functional and pain-related outcomes by fracture type and timing to further clarify differential trajectories following vertebral and non-vertebral fractures.

### 4.6. Alkaline Phosphatase and HbA1c

Alkaline phosphatase and HbA1c were primarily assessed as descriptive laboratory parameters to characterize bone turnover and metabolic status in this elderly real-life cohort. The observed decrease in the total cohort may reflect reduced bone turnover under antiresorptive therapy, age-related changes in bone metabolism, and BMI-dependent differences, as alkaline phosphatase is not a bone-specific marker but influenced by multiple tissues. Therefore, changes in alkaline phosphatase should be interpreted cautiously and in a descriptive context.

HbA1c levels showed only minor, non-significant changes over time, which is consistent with the absence of a direct metabolic effect of osteoporosis-specific pharmacological therapy. The slight downward trend observed may be related to indirect effects, such as improved mobility and increased physical activity resulting from pain reduction and functional stabilization. Increased physical activity is known to positively influence glucose metabolism in elderly population. However, given the observational design, these findings should be interpreted as hypothesis-generating rather than causal.

### 4.7. Strengths and Limitations

A major strength of this study is its real-life design, reflecting the association of guideline-based pharmacological osteoporosis therapy with clinically relevant outcomes under routine care conditions in a very elderly population. The comprehensive assessment of bone mineral density, laboratory markers, functional performance, and patient-reported pain outcomes allows evaluation beyond skeletal endpoints alone. The longitudinal study design and the use of linear mixed models further strengthen the robustness and validity of the findings.

However, several limitations must be acknowledged. The monocentric observational design and potential selection bias may limit generalizability of the results. In addition, the absence of an untreated control group precludes causal inference. Observed associations may be influenced by regression to the mean, co-interventions, survivorship bias and changes in physical activity or supportive care. In this study only serum calcium was assessed and monitored. Ionized calcium, which may provide additional information, was not measured in this real-life setting. Furthermore, the high prevalence of major osteoporotic fractures, particularly vertebral fractures, may limit generalizability to less severely affected populations. Given the exploratory nature of several secondary and tertiary outcomes and the absence of a global multiplicity adjustment, these results should be interpreted cautiously. The consistency of effects across multiple functional domains and the robustness of primary functional outcomes after FDR-based sensitivity analysis strengthen the credibility of the main findings. All in all, the results should be interpreted as hypothesis-generating and warrant confirmation in larger, preferably prospective, multicenter studies.

### 4.8. Conclusions

In this real-life cohort of elderly patients with osteoporosis, guideline-based specific pharmacological osteoporosis therapy was associated with significant improvements in bone mineral density, balance-related functional performance and pain outcomes under routine clinical care. Despite a concurrent decline in handgrip strength, the overall pattern suggests preserved or improved functional capacity in daily life.

These findings highlight that pharmacological therapy alone may be insufficient to counteract age-related muscle decline and should therefore be systematically combined with targeted muscle-strengthening and balance interventions.

Future research should include larger multicenter studies to enhance generalizability and to better identify subgroups with specific therapeutic needs or differential treatment responses. In addition, upcoming studies should evaluate combined treatment strategies, particularly the integration of pharmacological therapy, exercise interventions, and digital monitoring, to further optimize functional outcomes and fracture prevention.

The practical relevance of this study lies not in questioning the indication of SPOT, which is well established in current clinical guidelines, but in providing real-world evidence on its functional and pain-related associations in a very elderly and multimorbid population. While osteoporosis therapy is primarily prescribed to reduce the fracture risk and to improve the bone structure, our findings demonstrate that under routine care conditions it is also associated with improvements in balance-related performance, sit-to-stand ability, and back pain. Importantly, no relevant differences in functional or pain-related trajectories were observed between antiresorptive and osteoanabolic treatment groups. This suggests that, in advanced age, the choice of pharmacological agent should continue to be guided by fracture risk, prior fractures, and guideline recommendations. Importantly, the observed decline in handgrip strength despite concurrent improvements in balance-related performance, sit-to-stand ability, and pain-free mobility suggests that handgrip strength alone may underestimate functional muscular capacity in very elderly patients with osteoporosis. While handgrip strength is an established marker of sarcopenia, it predominantly reflects upper-limb strength and may be strongly influenced by age-related comorbidities, degenerative joint disease, and overall health status in this population. In contrast, the Chair Rise Test and tandem stance assess lower-limb strength, balance, and postural control, which are more directly related to mobility, fall risk and daily functional independence. The simultaneous improvement of these parameters together with increased pain-free walking and standing time indicates preserved or even enhanced functional performance in daily life, despite declining handgrip strength. Therefore, in very elderly and multimorbid patients with osteoporosis, a combined functional assessment using Chair Rise Test and tandem stance may provide a more comprehensive and clinically meaningful evaluation of muscular function and sarcopenia-related impairment than handgrip strength alone. These findings support the integration of multidimensional functional testing into routine osteoporosis care, particularly when monitoring therapy-associated changes beyond bone mineral density.

## Figures and Tables

**Table 1:** Overview of therapy groups and treatment duration.

Therapy Group	*N* (%)	Therapy Details	Median Therapy Duration
Antiresorptive Therapy total 142 (79.8%)			5 Years (Range: 1–24 Years)
Antiresorptive monotherapy	90 (50.6%)	Bisphosphonates 76	4 Years (Range: 1–20 Years)
		Denosumab 12	
		Raloxifen 1	
		Strontium ranelate 1	
Antiresorptive sequential therapy	52 (29.2%)	Sequential regimens	5 Years (Range: 1–24 Years)
		involving switches	
		between antiresorptive	
		agents	
Osteoanabolic Therapy total 36 (20.2%)			3 Years (Range: 1–23 Years)
Osteoanabolic monotherapy	6 (3.3%)	Teriparatide	1 Years (Range: 1–3 Years)
Osteoanabolic sequential therapy	30 (16.9)	Teriparatide with	3.5 Years (Range: 1–23 Years)
		preceding or subsequent	
		antiresorptive therapy	
Total cohort 178 (100.0%)			4 Years (Range: 1–24 Years)

**Table 2:** T-score under SPOT. T-scores at four time points (T0–T3) in the total cohort and stratified by sex. Data are presented as median + IQR + Range. A highly significant increase in T-score was observed over the observation period (β = 0.445; *p* < 0.001).

	T0	T1	T2	T3
T-Score Total cohort	−3.50 SD (IQR: 0.96; Range: −6.00–−2.50 SD)	−3.30 SD (IQR: 0.98; Range: −4.90–−1.60 SD)	−3.35 SD (IQR: 0.80; Range: −5.00–−1.20 SD)	−3.10 SD (IQR: 1.20; Range: −4.90–−0.90 SD)
T-Score Women	−3.55 SD (IQR: 0.95; Range: −6.00–−2.50 SD)	−3.33 SD (IQR: 0.96; Range: −4.90–−1.70 SD)	−3.40 SD (IQR: 0.78; Range: −5.00–−1.30 SD)	−3.15 SD (IQR: 1.15; Range: −4.90–−1.00 SD)
T-Score Men	−3.45 SD (IQR: 1.00; Range: −5.80–−2.60 SD)	−3.25 SD (IQR: 1.05; Range: −4.80–−1.60 SD)	−3.30 SD (IQR: 0.85; Range: −4.90–−1.20 SD)	−3.05 SD (IQR: 1.10; Range: −4.80–−0.90 SD)

**Table 3:** Serum 25(OH)D levels. Serum 25(OH)D concentrations at four time points (T0–T3) in the total cohort and stratified by sex. Data are presented as median + IQR + Range. A highly significant increase in 25(OH)D levels was observed over the observation period (β = 19.87; *p* < 0.001).

	T0	T1	T2	T3
Vitamin D Total cohort	69.4 nmol/L (IQR: 38.2; Range: 11.0–156.0 nmol/L)	75.7 nmol/L (IQR: 31.4; Range: 21.3–212.0 nmol/L)	78.6 nmol/L (IQR: 32.8; Range: 25.4–174.2 nmol/L)	83.5 nmol/L (IQR: 32.3; Range: 31.9–148.0 nmol/L)
Vitamin D Women	67.8 nmol/L (IQR: 36.4; Range: 11.0–154.0 nmol/L)	74.9 nmol/L (IQR: 30.2; Range: 22.0–208.0 nmol/L)	77.4 nmol/L (IQR: 33.0; Range: 25.4–174.2 nmol/L)	82.0 nmol/L (IQR: 32.1; Range: 33.0–148.0 nmol/L)
Vitamin D Men	73.0 nmol/L (IQR: 40.5; Range: 15.0–156.0 nmol/L)	77.5 nmol/L (IQR: 33.2; Range: 21.3–212.0 nmol/L)	81.0 nmol/L (IQR: 34.0; Range: 35.4–139.0 nmol/L)	85.0 nmol/L (IQR: 31.5; Range: 31.9–103.0 nmol/L)

**Table 4:** Serum calcium levels. Serum calcium concentrations at four time points (T0–T3) in the total cohort and stratified by sex. Data are presented as median + IQR + Range. A highly significant decrease in calcium levels was observed over the observation period (β = −0.047; *p* < 0.001).

	T0	T1	T2	T3
Calcium Total cohort	2.40 mmol/L (IQR: 0.16; Range: 1.92–3.30 mmol/L)	2.42 mmol/L (IQR: 0.17; Range: 2.20–3.02 mmol/L)	2.39 mmol/L (IQR: 0.125; Range; 2.01–2.91 mmol/L)	2.39 mmol/L (IQR: 0.14; Range: 2.08–3.10 mmol/L)
Calcium Women	2.41 mmol/L (IQR: 0.17; Range: 1.92–3.30 mmol/L)	2.42 mmol/L (IQR: 0.17; Range: 2.20–3.02 mmol/L)	2.39 mmol/L (IQR: 0.12; Range; 2.01–2.91 mmol/L)	2.39 mmol/L (IQR: 0.13; Range: 2.14–3.10 mmol/L)
Calcium Men	2.39 mmol/L (IQR: 0.10; Range: 2.15–2.69 mmol/L)	2.40 mmol/L (IQR: 0.10; Range: 2.27–2.60 mmol/L)	2.34 mmol/L (IQR: 0.12; Range: 2.27–2.60 mmol/L)	2.35 mmol/L (IQR: 0.085; Range: 2.08–2.50 mmol/L)

**Table 5:** Handgrip strength under SPOT. Handgrip strength of the dominant and non-dominant hand (in kg) at four time points (T0–T3) in the total cohort and stratified by sex. Data are presented as median + IQR + Range. A highly significant decline in dominant handgrip strength (β = −1.954; *p* < 0.001) and a significant decline in non-dominant handgrip strength (β = −0.830; *p* = 0.046) was observed over the observation period.

	T0	T1	T2	T3
Dominant handgrip strength				
Dominant handgrip strength Total cohort	26.0 kg (IQR: 10; Range: 9.0–62.0 kg)	26.0 kg (IQR: 11; Range: 6.0–61.0 kg)	25.50 kg (IQR: 11.0 kg; Range: 6.0–59.0 kg)	24.0 kg (IQR: 13.0; Range: 6.0–56.0 kg)
Dominant handgrip strength Women	24.0 kg (IQR: 7; Range: 9.0–50.0 kg)	24.0 kg (IQR: 9; Range: 6.0–49.0 kg)	24.0 kg (IQR: 8.75 kg; Range: 6.0–50.0 kg)	23.0 kg (IQR: 11.0; Range: 6.0–52.0 kg)
Dominant handgrip strength Men	38.0 kg (IQR: 13; Range: 14.0–62.0 kg)	36.0 kg (IQR: 15.0; Range: 17.0–61.0 kg)	36.0 kg (IQR: 14.0 kg; Range: 15.0–59.0 kg)	37.0 kg (IQR: 13.5; Range: 12.0–56.0 kg)
Non-dominant handgrip strength				
Non-dominant handgrip strength Total cohort	23.0 kg, (IQR: 8.0; Range: 4.0–57.0 kg)	24.0 kg (IQR: 10.0; Range: 6.0–56.0 kg)	24.0 kg (IQR: 10.0; Range: 6.0–57.0 kg)	22.0 kg (IQR: 10.0; Range: 8.0–48.0 kg)
Non-dominant handgrip strength Women	22.0 kg (IQR: 6.25; Range: 4.0–46.0 kg)	22.0 kg (IQR: 6.0; Range: 6.0–43.0 kg)	22.0 kg (IQR: 7.0; Range: 6.0–46.0 kg)	20.0 kg (IQR: 7.5; Range: 8.0–41.0 kg)
Non-dominant handgrip strength Men	33.0 kg (IQR: 10.0; Range: 14.0–57.0 kg)	34.0 kg (IQR: 12.0; Range: 14.0–56.0 kg)	34.0 kg (IQR: 14.0; Range: 12.0–57.0 kg)	26.0 kg (IQR: 9.0; Range: 12.0–48.0 kg)

**Table 6:** Chair Rise Test and tandem stance during SPOT. CRT and TS performance (seconds) at two time points (T0–T3) in the total cohort and stratified by sex. Data are presented as median + IQR + Range. CRT time improved significantly (β = −0.98; *p* = 0.004), and TS time increased significantly (β = +0.84; *p* = 0.007) over the observation period.

	T0	T3
Chair Rise Test		
Chair Rise Test Total cohort	11.0 s (IQR: 3.0; Range: 7–20 s)	10.0 s (IQR: 3.0; Range: 6–18 s)
Chair Rise Test Women	11.0 s (IQR: 3.0; Range: 7–19 s)	10.0 s (IQR: 3.0; Range: 6–18 s)
Chair Rise Test Men	12.0 s (IQR: 3.0; Range: 8–20 s)	11.0 s (IQR: 3.0; Range: 7–18 s)
Tandem stance		
Tandem stance Total cohort	9.0 s (IQR: 4.0; Range: 2–20 s)	10.0 s (IQR: 5.0; Range: 3–20 s)
Tandem stance Women	9.0 s (IQR: 4.0; Range: 2–18 s)	10.0 s (IQR: 4.0; Range: 3–19 s)
Tandem stance Men	10.0 s (IQR: 5.0; Range: 3–20 s)	11.0 s (IQR: 6.0; Range: 4–20 s)

**Table 7:** Pain parameters under SPOT. Back pain intensity, pain-free walking time, and pain-free standing time at two time points (T0–T3) in the total cohort. Data are presented as median + IQR + Range. A (highly) significant improvement in back pain intensity (β = −1.46; *p* < 0.001), pain-free walking time (β = +37.55; *p* = 0.031), and pain-free standing time (β = +31.2; *p* = 0.038) was observed over the observation period.

	T0	T3
Back pain		
Back pain Total cohort	5.0 NRS (IQR: 3.0; Range: 0–10 NRS)	3.0 NRS (IQR: 3.0; Range: 0–9 NRS)
Pain-free walking time		
Pain-free walking time Total cohort	60.0 min (IQR: 90.0; Range: 0–500 min)	99.0 min (IQR: 68.0; Range: 15–200 min)
Pain-free standing time		
Pain-free standing time Total cohort	45.0 min (IQR: 60.0; Range: 0–400 min)	78.0 min (IQR: 55.0; Range: 20–160 min)

**Table 8:** Serum concentrations of AP and HbA1c under SPOT. AP and HbA1c concentrations at two time points (T0–T3) in the total cohort and stratified by sex. Data are presented as median + IQR + Range. A significant decrease in AP levels (β = −0.137; *p* = 0.003) and a non-significant decrease in HbA1c levels (β = −0.063; *p* = 0.631) was observed over the observation period.

	T0	T3
Alkaline phosphatase		
Alkaline phosphatase Total cohort	1.34 µkat/L (IQR: 0.57; Range: 0.29–4.42 µkat/L)	1.28 µkat/L (IQR: 0.51; Range: 0.36–3.48 µkat/L)
Alkaline phosphatase Women	1.35 µkat/L (IQR: 0.58; Range: 0.29–4.42 µkat/L)	1.23 µkat/L (IQR: 0.51; Range: 0.36–3.48 µkat/L)
Alkaline phosphatase Men	1.28 µkat/L (IQR: 0.53; Range: 0.92–2.66 µkat/L)	1.32 µkat/L (IQR: 0.45; Range: 0.81–1.85 µkat/L)
HbA1c		
HbA1c Total cohort	5.70% (IQR: 0.77; Range: 4.80–10.0%)	5.80% (IQR: 0.36; Range: 4.90–9.00%)
HbA1c Women	5.70% (IQR: 0.60; Range: 5.10–8.20%)	5.90% (IQR: 0.50; Range: 5.10–9.00%)
HbA1c Men	5.80% (IQR: 1.00; Range: 4.80–10.00%)	5.70% (IQR: 0.20; Range: 4.90–6.30%)

**Table 9:** Overview of fracture locations per patient in the total cohort, categorized as MOF/other fractures/no fractures. Each fracture location was counted only once per patient to avoid duplicate entries. The majority of patients (*n* = 70; 39.3%) had at least one thoracic vertebral fracture.

Fracture Location	Number	Range
130 (=73.0%) patients with major osteoporotic fractures		
Proximal humerus	15 (=8.4%) patients	Range: 0–2
Distal radius	55 (=30.9%) patients	Range: 0–6
Proximal femur	12 (=6.7%) patients	Range: 0–1
Pelvis	10 (=5.6%) patients	Range: 0–1
Cervical spine	4 (=2.2%) patients	Range: 0–2
Thoracic spine	70 (=39.3%) patients	Range: 0–8
Lumbar spine	54 (=30.3%) patients	Range: 0–5
Sacral	3 (=1.7%) patients	Range: 0–1
91 (=51.1%) patients with other type of fractures		
Rib	28 (=14.8%) patients	Range: 0–4
Sternum	2 (=1.1%) patients	Range: 0–1
Hand	22 (=12.4%) patients	Range: 0–2
Foot	42 (=23.6%) patients	Range: 0–4
Other locations	39 (=21.9%) patients	Range: 0–3
24 (=13.5%) patients without any fractures		

**Table 10:** Overview of comorbidities per patient in the total cohort, categorized by pathophysiological groups. Each comorbidity category was counted only once per patient to avoid duplicate entries. The majority of patients (*n* = 106; 59.6%) presented with at least one endocrinology/metabolism-related comorbidity.

Comorbidity Category	Number of Patients
Endocrinology/metabolism	106 (=59.6%) patients
Cardiovascular	89 (=50.0%) patients
Pulmonology	17 (=9.6%) patients
Nephrology	28 (=15.7%) patients
Gastroenterology/liver/pancreas	15 (=8.4%) patients
Immunology/rheumatology	16 (=9.0%) patients
Neurology	9 (=5.1%) patients
Ophthalmology	3 (=1.7%) patients
Psychiatry	27 (=15.2%) patients
Other conditions	5 (=2.8%) patients

## Data Availability

The raw data supporting the conclusions of this article will be made available by the authors on reasonable request.

## Institutional Review Bord Statement

The study was conducted in accordance with the Declaration of Helsinki and approved by the Institutional Ethics Committee of the medical faculty of the University of Rostock on 25 February 2020 (approval number: A 2020-0041).

## Abbreviations

The following abbreviations are used in the manuscript:

%	Percent
25(OH)D	25-hydroxyvitamin D
AP	Alkaline phosphatase
BMI	Body mass index
BP	Back pain
CRT	Chair Rise Test
d	Cohen’s d
DXA	Dual-energy X-ray absorptiometry
DVO	Dachverband Osteologie (German Osteology Society)
EWGSOP2	European Working Group on Sarcopenia in Older People 2
FDR	False Discovery Rate
HbA1c	Glycated hemoglobin A1c
IQR	Interquartile range
kg	kilogram
LMM	Linear mixed model
m	meter
m^2^	Square meter
min	Minute
mmol/L	Millimole per liter
MOF	Major osteoporotic fracture(s)
Nmol/L	Nanomole per liter
NRS	Numerical Rating Scale
PAD	Peripheral arterial disease
PFST	Pain-free standing time
PFWT	Pain-free walking time
REML	Restricted maximum likelihood
s (sec)	Second
SD	Standard deviation
SE	Standard error
SPOT	Specific pharmacological osteoporosis therapy
SPSS	Statistical Package for the Social Sciences
TS	Tandem stance
WHO	World Health Organization
χ^2^	Likelihood ratio test
β	Effect coefficients
µkat/L	Microkatal per liter
